# New Robust Face Recognition Methods Based on Linear Regression

**DOI:** 10.1371/journal.pone.0042461

**Published:** 2012-08-07

**Authors:** Jian-Xun Mi, Jin-Xing Liu, Jiajun Wen

**Affiliations:** 1 Bio-Computing Research Center, Shenzhen Graduate School, Harbin Institute of Technology, Shenzhen, Guangdong Province, China; 2 Key Laboratory of Network Oriented Intelligent Computation, Shenzhen, Guangdong Province, China; Institute of Psychology, Chinese Academy of Sciences, China

## Abstract

Nearest subspace (NS) classification based on linear regression technique is a very straightforward and efficient method for face recognition. A recently developed NS method, namely the linear regression-based classification (LRC), uses downsampled face images as features to perform face recognition. The basic assumption behind this kind method is that samples from a certain class lie on their own class-specific subspace. Since there are only few training samples for each individual class, which will cause the small sample size (SSS) problem, this problem gives rise to misclassification of previous NS methods. In this paper, we propose two novel LRC methods using the idea that every class-specific subspace has its unique basis vectors. Thus, we consider that each class-specific subspace is spanned by two kinds of basis vectors which are the common basis vectors shared by many classes and the class-specific basis vectors owned by one class only. Based on this concept, two classification methods, namely robust LRC 1 and 2 (RLRC 1 and 2), are given to achieve more robust face recognition. Unlike some previous methods which need to extract class-specific basis vectors, the proposed methods are developed merely based on the existence of the class-specific basis vectors but without actually calculating them. Experiments on three well known face databases demonstrate very good performance of the new methods compared with other state-of-the-art methods.

## Introduction

Face recognition, a user-friendly identity authentication technology, has become one of the most intensively studied topics in computer science [1]–[3], and is also very popular in neuroscience [4], [5], which has a lot of important applications in security systems, law enforcement, and commerce. Two main issues in a face recognition system are feature extraction and classification. Early researchers used geometric features of a face to perform recognition. However, studies showed that template matching methods outperform the geometric feature-based ones. Therefore, appearance-based method became the mainstream [6]. However, the dimension of the original images is usually very high. To avoid the curse of dimensionality, extracted features from face images are used to perform classification in a low dimensional feature space [7]. The widely used linear feature extraction approaches include Principle Component Analysis (PCA) [8], Independent Component Analysis (ICA) [9] and Linear Discriminant Analysis (LDA) [10], etc. However, recent studies suggested that even simple features produced by the methods, such as downsampling and random projection, can work as well as others [11], [12]. Therefore, to design a robust classifier is of the key importance for the face recognizer.

Classification using linear regression-based technique is a straightforward strategy to recognition the unknown faces. Recently, many such methods are proposed. In Nearest Feature Space (NFS) classifier, also known as Nearest Subspace classifier, first proposed in [13], samples from each individual class are combined to form a class-specific model [12] of its class-specific space. The test image is assigned to the class which has the minimum regression error. To improve the performance of NS, very sophisticated features were used to develop the feature space in early work [13]. A recent extension work of NS classification, namely LRC [12], uses the downsampled images as features for recognition, which achieves fairly high accuracy. In opposite to NS methods, in [14] the test sample is represented by all training samples, which means the entire training set is used as a linear model to predict the probe image. A two-phase global representation method was proposed in [3] where the double representation of the test images increases the recognition accuracy. Similar to [14] and [3], the selected training samples across classes are used to perform global representation of the probe [7]. The Sparse Representation-based Classification (SRC) method can also be viewed as a linear regression-based approach in a sense [11], however, sparsity constraint is imposed on the model parameters.

An importance assumption behind some popular face recognition methods [10]–[12], [15], such as LRC and SRC, is that the samples belonging to an individual class tend to lie on a class-specific subspace. A proof that face images of a class under various lighting is in a linear subspace is given in [16]. In this paper, we propose a new idea that the effectiveness of LRC also comes from the basis vectors of each class-specific subspace, i.e. class-specific basis vectors which have been used in previous studies. In [17], eigen-decomposition of each class-specific subspace is conducted to find class-specific basis vectors. A similar method using concept of the common vector is introduced in [18] (note that the common vectors are different from our common basis vectors). Then very efficient algorithms were proposed in [19] to perform face recognition, which used an orthonormal optimal projection matrix to obtain the common vector of an individual class. A recent work proposed a kernel common vector method to handle the nonlinearity [20].

With the concept of class-specific basis vectors, we consider a class-specific subspace is spanned by two kinds of basis vectors: The first one is the class-specific basis vector, also known as the discriminative vector for each class, owned by only one class; the second one is common basis vector shared by several classes. In this sense, we have a new explanation of the effectiveness of LRC. That is, the class to which the test sample belongs still has the minimum regression error is because the subspace of this class contains the class-specific basis vectors while other classes can only provide common basis vectors. In general, a complete class-specific subspace should consist of many common basis vectors, because there are a great number of variations due to illumination, facial expressions and pose variations etc. However, face recognition is an SSS problem in general [21], thus it is not surprise that some common basis vectors are absent for a certain class. More importantly, it is hard to predict which common basis vectors are absent, so that for one class certain common basis vectors may be absent, however, which could be included in other classes. For this reason, the linear regression becomes unstable, which reduces the recognition performance of previous NS methods.

**Figure 1:**
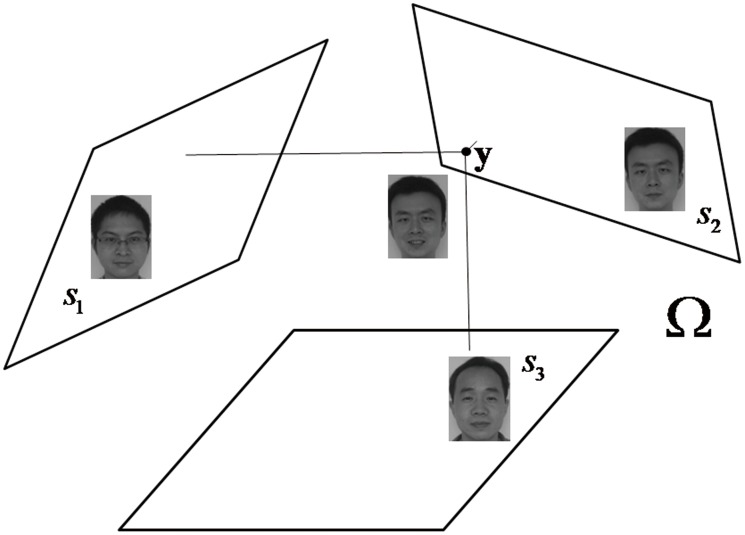
A demonstration of three class-specific subspaces embedded in sample space. 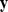
 is a test image from class 2.

To build a classifier robust against the SSS problem, we propose a novel linear model, called the “leave-one-class-out” subspace model. For one class, its “leave-one-class-out” subspace consists of all the common vectors and class-specific basis vectors for other classes but does not include any class-specific basis vectors of itself. Hence, distances between a test image and “leave-one-class-out” subspaces are capable of providing important discrimination information. By using the “leave-one-class-out” subspace, we develop two new linear regression-based classification methods, i.e. RLRC 1 and 2, to give a robust classification of test images. The discriminative information of RLRC 1 totally depends on the “leave-one-class-out” subspace, whereas RLRC 2 fuses discriminative information both from the “leave-one-class-out” subspace and the class-specific subspace. These two new methods can reduce the misclassification rate caused by loss of the common basis vectors. Thus it is more robust than the original LRC. The good performance of our methods is shown in experiments.

**Figure 2:**
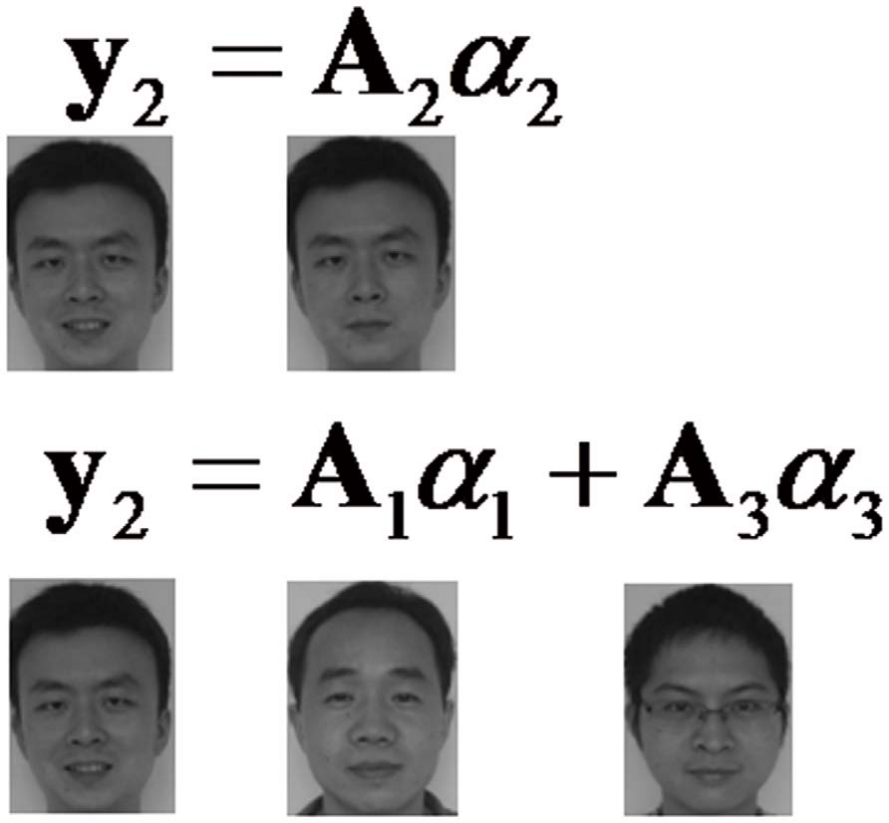
An illustration which demonstrates a test image 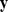 can be represented by model 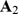 or by model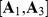.

Three main contributions of this paper are as follows. Firstly, we analyze the reason why the NS classifier can work effectively. Secondly, we build a “leave-one-class-out” subspace for each class as a linear model which makes better use of class-specific basis vectors. Thirdly, by using the new subspaces, we proposed two novel classification methods which achieve promising recognition accuracy. Unlike the studies which actually extract discriminative vectors of each class [17], [19], our method exploit the concept of the class-specific basis vectors but without calculating them. Therefore, the proposed methods are more efficient.

**Figure 3:**
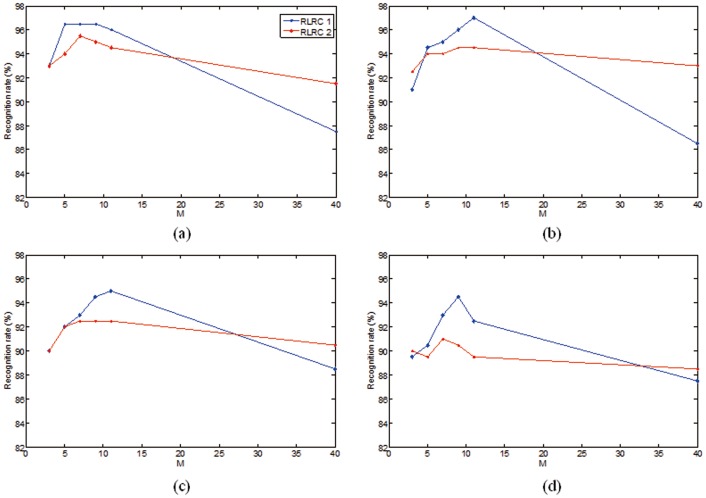
Face recognition rates of RLRC1 and RLRC2 on AT&T database. (a)–(b) The results for different image sizes, which are 40×40, 30×30, 20×20, and 15×15, respectively.

## Materials and Methods

Let us present a typical face recognition scenario. Consider there are 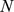 distinguished classes and 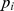 prototype images for the 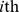
class, 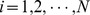. Each prototype will be represented in feature space as a vector 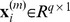, where 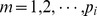 and 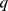 is the dimension of feature space for all the images. We model the class-specific subspace by stacking the training vectors of the 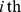 class. Then the developed class-specific model of the 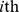 class is denoted as
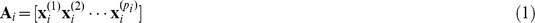
where 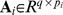. Many studies suggest that the samples belonging to a certain class lie on their class-specific subspace. Therefore, a test image 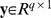, belonging to the 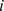 th class, should satisfy the following equation approximately:
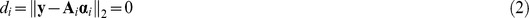
where 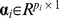 is the parameter vector of the 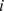
th class model. That is to say, 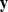 lies on or is closed to the subspace of its own class. While the subspaces of other classes have longer distances from 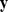, i.e. 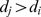, where 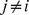,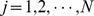. Therefore, NS classification considers the test image comes from the nearest class-specific subspace. From the other viewpoint, NS classification seeks to find a class-specific model which could give a test image the best prediction so that the task of face recognition can be defined as a problem of linear regression. The parameter vector of each model is calculated by using least-squares estimation [12]:
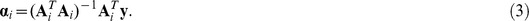
where 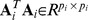 is well conditioned since there are few samples within each class and the feature dimension is usually high. Using the estimated parameter vectors, 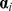, we can calculate the predicted vector by using the 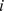
th class model
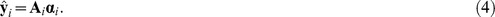


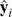
 can also be viewed as the projection of 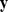 on to the 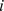
th subspace. By comparing the predicted vector and the original test image in the Euclidean sense,
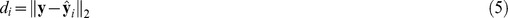
we rule in favor of the class which has minimum 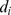. Note that the LRC algorithm, proposed in [12], is an improved version of NS classification method using the downsampled images as features.

**Table 1:** A comparison of face recognition rates on AT&T Database.

Approach	Recognition Rate						
	40×40	30×30	20×20	15×15	*ε* = 0.1	*ε* = 0.2	*ε* = 0.3
RLRC 1	**96.5%** (9)	**97.0%** (11)	**95.0%** (11)	**94.5**% (9)	/	/	/
RLRC 2	95.5% (7)	94.5% (11)	92.5% (11)	91.0% (7)	/	/	/
LRC	92.5%	91.5%	89.50%	88.5%	/	/	/
SRC	/	/	/	/	86.0%	91.0%	87.5%

The best results obtained by our method are listed together with the corresponding 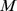 shown in brackets.

**Table 2:** A comparison among several methods on AT&T Database.

Approach	Fisherfaces	ICA	2DPCA	RLRC 1	RLRC 2	LRC	SRC	Kernel Eigenfaces
Recognition Rate	94.5%	85.0%	96.0%	**97.0%**	95.5%	92.5%	91.0%	94.0%

The best results are listed.

### Analysis of the Effectiveness of NS Classifier

The class-specific subspace is known to be embedded in the complete feature space spanned by all the samples [12], i.e. the sample space. Applying the idea of NS to face recognition is based on an assumed concept that samples from an individual class are on a class-specific subspace. However, there can be several possible explanations of this concept. One explanation is that each class-specific subspace has a separate location in the sample space. Figure 1 gives a simple illustration of this explanation where three face subspaces of 2D, i.e. 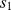,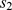, and 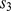, are embedded in a 3D complete sample space 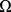. In this example, each of the three classes has its own class-specific subspace located in different position of 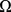. If there is a test sample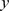, which belongs to class 2, NS method tries to find the nearest subspace of 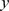, i.e. 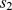. In other words, the linear model of the class 2, 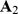, can give the most precise prediction of 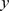. However, if we use a combined model 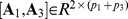 to predict 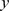, an accurate prediction can also be obtained, which is illustrated in Figure 2. Because the complete sample space 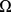 could be spanned by only using basis vectors of class 1 and class 3.

**Figure 4:**
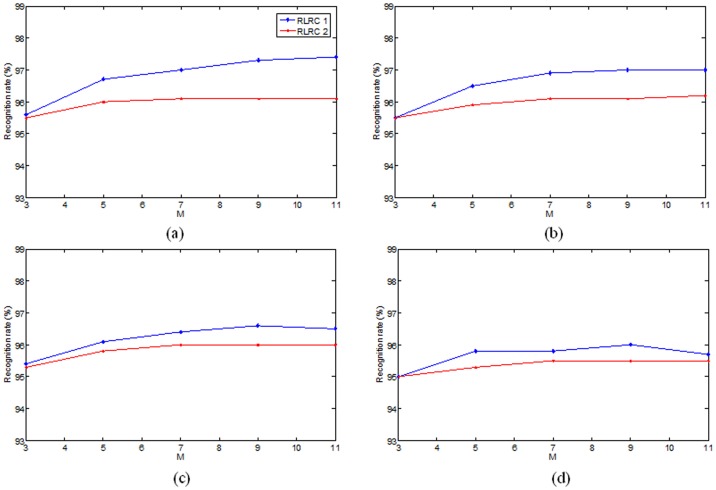
Face recognition rates of RLRC1 and RLRC2 on Extended Yale B database. (a)–(b) The results for different image sizes, which are 40×40, 30×30, 20×20, and 15×15, respectively.

However, the above description of class-specific subspaces does not coincide with our investigation that a sample cannot be modeled accurately by samples from others. It means that there must be some unique basis vectors owned by each class. In other words, a class-specific subspace consists of basis vectors of two different categories: The first are *the common basis vectors* shared by several classes. The second are *the class-specific basis vectors* owned by one class only. Here we give the mathematical description of this concept. Suppose the 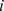 th class has 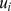 common basis vectors and 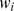 class-specific basis vectors. Then we define the 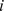 th class-specific subspace as
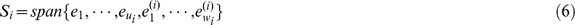
where 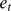,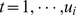, is a common basis vector and 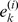,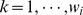, is a class-specific basis vector.

Next we will show how NS works if the class-specific subspace is spanned by two kinds of basis vectors. Without loss of generality, assume there is a test sample 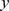, belonging to class 1, which lies in 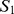:
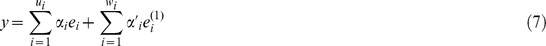
where 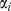 and 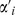 are the coordinates. Since without the class-specific basis vectors of class 1, 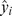 (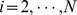), the prediction of 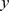 by other subspace, has a longer distance to 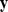 than 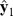, i.e.



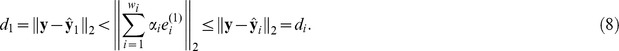
As a result, NS classifies 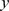 to the correct class. Hence, the above description is another explanation of the effectiveness of NS method. In next Section, we will present two new classification methods which will validate the existence of the common basis vectors and the class-specific basis vectors.

**Table 3:** A comparison of face recognition rates on Extended Yale B database.

Approach	Recognition Rate						
	40×40	30×30	20×20	15×15	*ε* = 0.1	*ε* = 0.2	*ε* = 0.3
RLRC 1	**97.4%** (11)	**97.0%** (11)	**96.6**% (9)	**96.0%** (9)	/	/	/
RLRC 2	96.1% (11)	96.2% (11)	96.0% (11)	95.5% (11)	/	/	/
LRC	95.0%	95.1%	94.6%	94.0%	/	/	/
SRC	/	/	/	/	**97.5%**	94.8%	86.7%

**Table 4:** A comparison among several methods on Extended Yale B database.

Approach	PCA+NN	LDA+NN	Laplacian+NN	RLRC 1	RLRC 2	LRC	SRC
Recognition Rate	88.4%	87.6%	90.7%	97.4%	96.2%	95.0%	**97.5%**

In many applications, face recognition is a SSS problem in a high dimensional space. Furthermore, since there are wide-range variations on human faces due to illumination, pose, viewpoint, etc, a small number of training samples for each class hardly span the complete class-specific subspace. It is very likely that, for a class, some of the common basis vectors are missing. In this case, if we use NS to perform classification, the classifier might misclassify the test samples. Here, we give an example following the one in previous paragraph to show the possible misclassification. Suppose the subspace 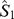 spanned by samples of class 1 lacks some common basis vectors. Therefore,
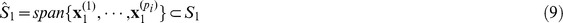
and
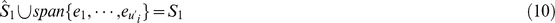
where 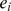,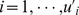, denotes an absent common basis. The distance between 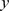 and the projection of 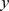 on 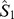 is
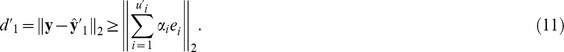
Other class-specific subspaces might have those common basis vectors, so that it is possible that 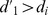. Therefore, the previous NS method is not robust enough to the SSS problem.

Now, we introduce two novel linear regression-based classification methods, i.e. RLRC 1 and 2.

**Table 5:** A comparison of face recognition rates using images without occlusion from AR database.

Approach	Recognition Rate						
	40×40	30×30	20×20	15×15	*ε* = 0.1	*ε* = 0.2	*ε* = 0.3
RLRC 1	**75.4%** (11)	74.2% (11)	74.6% (7)	72.1% **(**11)	/	/	/
RLRC 2	73.4% (11)	**74.3%** (9)	**74.7%** (9)	**75.2%** (11)	/	/	/
LRC	70.6%	70.9%	71.6%	71.0%	/	/	/
SRC	/	/	/	/	70.0%	71.9%	65.4%

### Robust LRC 1 Method

To design RLRC 1, we use the class-specific basis vectors owned by each class as the only discriminative information. First of all, note that compared with some previous studies which intended to extract the class-specific basis vectors of each class as the discriminative vectors, RLRC 1 exploits the existence of class-specific basis vectors but without actually calculating them. The core of our method is the “leave-one-class-out” model of a class, which consists of all the common basis vectors but does not include the class-specific basis vectors.

Let us give a brief introduce of the “leave-one-class-out” model. Without loss of generality, we suppose there is a test image 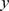 belonging to class 1. The “leave-one-class-out” model of class 1 is denoted as 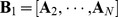. We can see 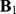 is exclusive of the class-specific basis vectors of class 1 and should include all the common basis vectors. This is because: First, the common basis vectors are shared by many classes, which means any common basis vector should be included in 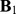; Second, the subspace of 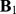 is a union of all the class-specific subspace except that of class 1, which ensures that any class-specific basis vectors of class 1 are excluded from 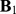.

And we develop 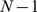 “leave-one-class-out” models for the rest classes, i.e. 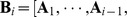 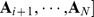, 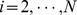, respectively. It is easy to find out that each subspace of “leave-one-class-out” model should include all the common basis vectors and also contains class-specific basis vectors of class 1. For this reason, if we use 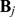
(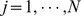) to model 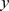, 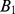 will produce the maximum residual error. Note that, for 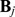 provides a helpful discrimination while avoiding the problem of missing common basis vectors that happened in previous NS methods. In summary, we use 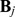 to model the test sample, and this test sample is classified to the class which has the maximum residual error. We refer to the above described approach as the “leave-one-class-out” scheme.

However, since there are always noises in sample collection, 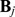 will overfit the test image if too many classes are included in 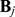. Here we propose a scheme to reduce the number of classes the basic idea of which is that we use the distance between the test sample and each class-specific subspace to determine 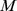 classes which are very likely to involve the class to which the test sample belongs. Therefore, the first step of RLRC 1 is to select the first 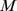 classes according to the ascending order of distances between the test image and class-specific subspaces 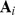. Here, we use an index set 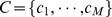 to denote the labels of the 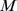 selected classes, in which the element 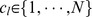 (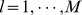) is the class label. For example, if 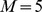 and 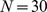, a possible case is 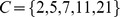
_._ The second step of RLRC 1 is to conduct “leave-one-class-out” scheme using the training samples from the selected classes. First we need to define the “leave-one-class-out” model:
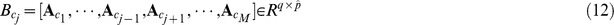
where 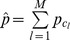, and 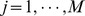. Then we use 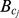 to estimate the predication of the test image 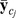 by the parameter vector 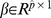, i.e.
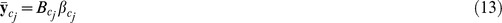
and
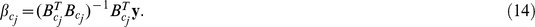
Next, we measure the distance between 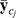 and 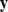,
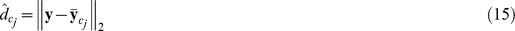
Finally, we classify the test sample to the class having maximum 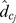. The complete recognition procedure is summarized in Algorithm 1.

**Table 6:** Recognition rates for Occluded faces by sunglasses.

Approach	Recognition Rate						
	40×40	30×30	20×20	15×15	*ε* = 0.1	*ε* = 0.2	*ε* = 0.3
RLRC 1	**99.0%** (9)	**99.0%** (9)	97.5% (9)	94.0% (9)	/	/	/
RLRC 2	**99.0%** (9)	98.5% (9)	**98.5%** (9)	**98.5%** (11)	/	/	/
LRC	**99.0%**	98.5%	98.0%	97.0%	/	/	/
SRC	/	/	/	/	96.0%	97.0%	96.5%

### Algorithm 1 The Robust Linear Regression Classification 1 (RLRC 1)

**Inputs:** Linear models 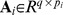, 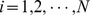
_,_ and a test sample 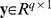.

**Output:** Class label of **y**

1. Compute the parameter vector for model 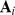: 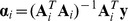, 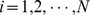.
2. Calculate the residuals 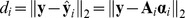.
3. Select the first 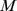 minimum 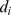 and record their indices in 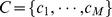.
4. Build 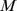 “leave-one-class-out” models, 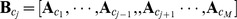,
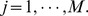

5. Compute the parameter vector of model 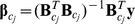.
6. Calculate the residuals 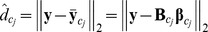
_._
7. Decision is made in favor of the class having the maximum 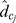,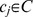.

**Table 7:** A comparison among several methods for Occluded faces on AR database.

Approach		PCA+NN	ICA 1+NN	LNMF+NN	RLRC 1	RLRC 2	LRC	SRC
Recognition Rate	Sunglasses	70.0%	53.5%	33.5%	**99.0%**	**99.0%**	**99.0%**	97.0%
	Scarf	12.0%	15.0%	24.0%	44.0%	40.5%	33.5%	**67.5%**

**Table 8:** Recognition rates for Occluded faces by Scarf.

Approach	Recognition Rate						
	40×40	30×30	20×20	15×15	*ε* = 0.1	*ε* = 0.2	*ε* = 0.3
RLRC 1	**44.0%** (11)	**38.0%** (11)	**34.5%** (11)	29.5% (9)	/	/	/
RLRC 2	40.5% (11)	37.0% (11)	33.0% (11)	**30.0%** (11)	/	/	/
LRC	33.5%	24.5%	29.0%	25.0%	/	/	/
SRC	/	/	/	/	**67.5%**	63.0%	51.0%

### Robust LRC 2 Method

Unlike RLRC 1 which only takes advantage of the class-specific basis vectors, we propose another algorithm, namely RLRC 2, which utilizes two kinds of discriminative information: distances between a test sample and class-specific subspaces 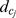 (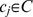) (in the second step of RLRC 1) and distances between a test sample and “leave-one-class-out” subspaces 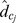. Our motivation of proposing RLRC 2 is to test whether these two kinds of discriminative information are different. To this end, we propose a simple fusion scheme to use 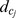 and 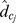 in RLRC 2. The new decision variable is defined as
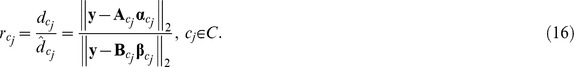



As mentioned before, the test sample should have small distance to the subspace of its own class, which means the test sample is very likely from the class having small 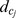. On the other hand, the test sample is considered to come from the class whose “leave-one-class-out” model has very large regression residual which means 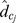
is large. Therefore, the final decision is to classify the test image to the class with minimum 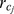. The complete recognition procedure is summarized in Algorithm 2.

### Algorithm 2 The Robust Linear Regression Classification 2 (RLRC 2)

**Inputs:** Linear models 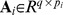,
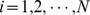
_,_ and a test sample 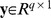.

**Output:** Class label of **y**

1. Compute the parameter vector for model 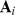: 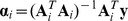, 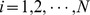.
2. Calculate the residuals 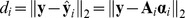.
3. Select the first 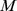
minimum 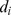 and record their indices in 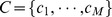 and the values 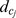,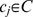.
4. Build 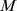 “leave-one-class-out” models, 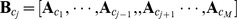, 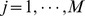.
5. Compute the parameter vector of model 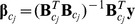.
6. Calculate the residuals 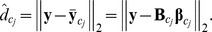

7. Compute the decision variables 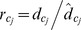, and decision is made in favor of the class having the minimum 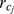,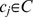.

Note that when 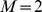, RLRC 1 and 2 methods are actually identical to NS method. Therefore, our new methods should be viewed as a generalized version of NS method. When we increase
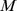
_,_ the recognition rates of our methods will increase compared to NS method. We will test our method with various 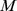 in experiments.

## Results

In this section, we test our methods on three benchmark face databases to demonstrate the performance of RLRC 1 and 2. 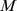
is the only parameter in our algorithms and we test algorithms with 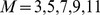 and total number of subjects on one database (only on AT&T database). Since we focus on the classifiers, downsampled gray images are used as features so as to compare with the latest NS method, i.e. LRC. We test the images with four different sizes: 40×40, 30×30, 20×20, and 15×15. Besides LRC, SRC is another method that used for comparison. It is because SRC is a very popular method sharing the same assumption on the face subspace with NS method. Showing good performance for face recognition [11], l1-magic tool is used to implement the sparse constraint in SRC. Due to noise existing in collection of the samples, the test sample is expressed approximately by the training samples from the right class. So, the stable l1-minimization problem is solved to obtain the sparse coding vector which is given by
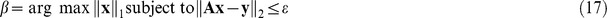
where a parameter 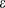 controls the sparsity of the solution. If 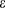 becomes small, which means the test sample is represented more precisely, and the coding vector 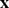 is less sparse. And if 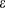 is big, less samples are required to represent the test sample so that 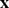 becomes sparse. We conduct the testing of SRC by setting 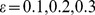, respectively. A random projection matrix 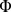 is also required in SRC [11], [22].

### AT&T Database

The AT&T database consists of 40 subjects with 10 images per subject [23], [24]. The images incorporate several variations, such as expression variations, wearing glasses or not. We implement the evaluation protocol following the previous studies [25], [26] which used the first five images of a subject as training samples, while the rest five are served as test samples. For there are 200 training samples, we let 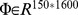 for SRC. The results are shown in Figure 3 and Table 1.

From Figure 1, it can be seen that our algorithms have good performance when 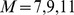. Therefore, the appropriate increase of 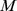 is beneficial to improve the recognition rate. However, when we let 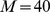 which means the entire training set is used and no class is removed before construction of “leave-one-class-out” models, the recognition rate decreases. This is because “leave-one-class-out” models overfit test images. We can see both RLRC 1 and 2 outperform LRC, which is shown in Table 1. SRC achieves the accuracy of 91.0% with 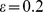 which is 6% and 4.5% worse than RLRC 1 and 2 respectively. Table 2 depicts a detailed comparison of our test methods with a variety of approaches reported in [27], consisting of Fisherfaces, ICA, Kernel Eigenfaces, and 2DPCA. Among all above mentioned methods, RLRC 1 obtains the highest recognition rate, up to 97%.

### Extended Yale B Database

The Extended Yale B database contains about 2,141 face images of 38 subjects, which incorporates varying illumination conditions [16], [28], [29]. Here, the cropped images are used in our experiment. One half of images of a subject are randomly selected as training samples, the rest half are served as test samples. Let 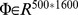 for SRC. Figure 4 shows the mean recognition rates of our algorithm over ten trials, and Table 3 and 4 show the comparisons.

From Figure 4, we can see proposed methods work well when 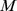 is set to 9 and 11. Table 3 shows that RLRC 2 outperforms LRC at least 1.1% and RLRC 1 achieves higher recognition rate, more than 2% improvement over LRC. RLRC 1 achieves almost an equal result comparing to SRC. When compared with the results of other classical methods, including PCA, LDA and Laplacian method (reported in [11]), our methods and recent LRC and SRC are more competitive.

### AR Database

We use cropped images of 50 males and 50 females from the AR database [30], [31]. Three experiments are conducted on this database. In the first experiment, seven images (without occlusion) of each subject from Session 1 are used for training, and other seven images from Session 2 are used for testing. We let 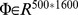 for SRC. For saving space, we will not show the results of our methods with different 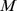. The best results of our methods are obtained when 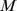
is not very small (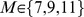 typically) as shown in the previous experiments. In Table 5, we can see that RLRC 1 has the highest recognition accuracy when the resolution of the images is 40×40. But using lower dimension features, RLRC 2 has better performance than RLRC1. The reason is that there are many subjects in AR database so that more class-specific basis vectors are required. If we reduce the dimension of feature space, the class-specific basis vectors become insufficient, which leads to weaker discriminative capability of RLRC1. Note that LRC and SRC are 3% behind at least.

The next two experiments are to study the performance of our methods against face occlusions including sunglasses and scarf. Eight images per subject (the first 4 images from Session 1 and 2) are used for training, while two images with sunglasses occlusion and two images with scarf occlusion are used for test [12]. Table 6 and 7 list the results for sunglasses. Three linear regression-based methods attain a very high recognition rate, up to 99.0%. Note that with lower dimension features, RLRC 2 obtains better results. The best recognition rate of SRC is 97% which lags 2% behind that of our methods. The PCA, ICA 1, and LNMF [32] with nearest neighbor (NN) classifier give low recognition rates of 70.0%, 53.5% and 33.5%, respectively [12]. For the case of scarf occlusion, we can see from Table 7 and 8 that although RLRC 1 outperforms LRC by 11.5%, SRC achieves the highest recognition accuracy of 67.5%, which means SRC is more robust to the big occlusions. The PCA, ICA 1, and LNMF attain very low recognition rates of 12.0%, 15.0% and 24.0%, respectively.

## Discussion

Here, we want to discuss the experimental results and present some conclusions:

1. RLRC 1 can perform face recognition very well, which is reliable evidence to validate that the class-specific basis vectors could provide strong discriminative information, even if we do not actually calculate them.
2. The performance of either RLRC 1 or RLRC 2 is different from that of LRC, which indicates that the discriminative information of the class-specific basis vectors is different from that originated from the location information of each class-specific subspace in sample space.
3. RLRC 1 has better performance in high-dimension feature space. The reason must be that the high-dimension feature space consists of rich class-specific basis vectors which will be beneficial for RLRC 1.
4. The parameter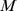 for RLRC 1 and 2 is not sensitive to data sets. Our methods obtain promising results on different face databases when the parameter is set to around 10.
5. RLRC 1 and 2 are a generalization of LRC method. When 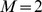, three methods are identical. And, from Figure 3 and 4, we can see that the results of RLRC 1 and 2 are close to LRC when 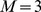. But the gap will widen together with the increase of 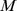.

### Conclusions

In this paper, we have contended theoretically and experimentally that class-specific basis vectors could provide very useful discriminative information to perform robust linear regression-based classification of face images. The “leave-one-class-out” subspace model which we use to build linear regression-based classification methods is more robust than the class-specific subspace model used in NS method. Next, two robust LRC methods are proposed by exploiting the existence of class-specific basis vectors. RLRC 1 uses the class-specific basis vectors as only discriminative information and RLRC 2 uses two different kinds of discriminative information. In our opinion, both RLRC 1 and 2 are generalizations of NS method. Furthermore, the proposed methods are computationally efficient, since they only need to solve linear regression problems. Finally, excellent discrimination capabilities of our methods are demonstrated by the experiments.
